# Soil ammonia-oxidizing archaea in a paddy field with different irrigation and fertilization managements

**DOI:** 10.1038/s41598-021-93898-y

**Published:** 2021-07-15

**Authors:** Limin Wang, Dongfeng Huang

**Affiliations:** 1 Soil and Fertilizer Institute, Fujian Academy of Agricultural Sciences, Fuzhou, 350012 People’s Republic of China; 2 Fujian Key Laboratory of Agro - Products Quality and Safety, Fujian Academy of Agricultural Sciences, Fuzhou, 350000 People’s Republic of China

**Keywords:** Microbiology, Environmental sciences

## Abstract

Because ammonia-oxidizing archaea (AOA) are ubiquitous and highly abundant in almost all terrestrial soils, they play an important role in soil nitrification. However, the changes in the structure and function of AOA communities and their edaphic drivers in paddy soils under different fertilization and irrigation regimes remain unclear. In this study, we investigated AOA abundance, diversity and activity in acid paddy soils by a field experiment. Results indicated that the highest potential ammonia oxidation (PAO) (0.011 μg NO_2_^-^ –N g^-1^ d.w.day^-1^) was found in T_2_ (optimal irrigation and fertilization)—treated soils, whereas the lowest PAO (0.004 μg NO_2_^-^ –N g^-1^ d.w.day^-1^) in T_0_ (traditional irrigation)- treated soils. Compared with the T_0_—treated soil, the T_2_ treatment significantly (*P* < 0.05) increased AOA abundances. Furthermore, the abundance of AOA was significantly (*P* < 0.01) positively correlated with pH, soil organic carbon (SOC), and PAO. Meanwhile, pH and SOC content were significantly (*P* < 0.05) higher in the T_2_—treated soil than those in the T_1_ (traditional irrigation and fertilization)- treated soil. In addition, these two edaphic factors further influenced the AOA community composition. The AOA phylum *Crenarchaeota* was mainly found in the T_2_—treated soils. Phylogenetic analysis revealed that most of the identified OTUs of AOA were mainly affiliated with *Crenarchaeota*. Furthermore, the T_2_ treatment had higher rice yield than the T_0_ and T_1_ treatments. Together, our findings confirm that T_2_ might ameliorate soil chemical properties, regulate the AOA community structure, increase the AOA abundance, enhance PAO and consequently maintain rice yields in the present study.

## Introduction

The oxidation of ammonia to nitrite was the first step in the soil nitrification process, which was driven by ammonia-oxidizing bacteria (AOB) and archaea (AOA)^1,2^, but their relative contributions to ammonia oxidation in different types of soils were still under debate^3–6^. Some researches showed that AOA might make more contributions than AOB in microbial ammonia oxidation through the enzyme ammonia monooxygenase (*amoA*)^3,4^. Other reports found that AOB played a more critical role than AOA^5,6^. In general, these two ammonia oxidizers played distinct roles in ammonia oxidation process under different soil and crop management systems, because they had different ecological niche partitioning^7^. In this study, the PCR amplification products of soil DNA samples were analyzed by 2% agarose gel electrophoresis. Results indicated that a bright band of the target DNA for only AOA was visible in the paddy soil.

The subtropical paddy soil was classified as typical Hapli-Stagnic Anthrosols characterized by low nutrient capital, low pH value, high P fixation that severely constrained rice production^8^. Rice production must, however, increase by 1% annually because of an increase in the population^9^. High rice yields mainly depend on higher inputs of nitrogen (N) and phosphorus (P) fertilizers, which led to soil quality deterioration. Therefore, the optimizal water and fertilizer management was commonly used to improve soil properties, which played a positive role in regulating AOA community structure^10,11^. Many reports have revealed that soil organic carbon (SOC), pH, nitrate N (NO_3_^-^ -N), ammonium N (NH_4_^+^-N) and their interactions could be responsible for shaping AOA composition^3,12^. Meanwhile, the AOA-driven potential ammonia oxidation (PAO) exhibited a strong response to soil property change associated with water supply and fertilizer input^13^. However, Fang et al. (2019) showed that no edaphic properties had an influence on AOA composition^10^. Overall, as the edaphic factors controlling AOA distribution in soils are highly heterogeneous in different studies, the heterogeneous soil environments have various effects on AOA community^14,15^. Hence, the relationships between the abundance, activity and composition of AOA communities and soil property changes were still needed to further investigated in this study. Here, we hypothesized that the abundance and composition of AOA community would be impacted by soil factors associated with fertilization and irrigation in an acid typic Hapli-Stagnic Anthrosol across southeastern China; such an AOA shift may further influence PAO activity. To verify the hypothesis, a field experiment was conducted to estimate the soil chemical properties, AOA abundance, activity, and community composition in soils under different fertilization and irrigation regimes.

## Results

### Edaphic characteristics, potential ammonia oxidation, and rice yields

**Figure 1:**
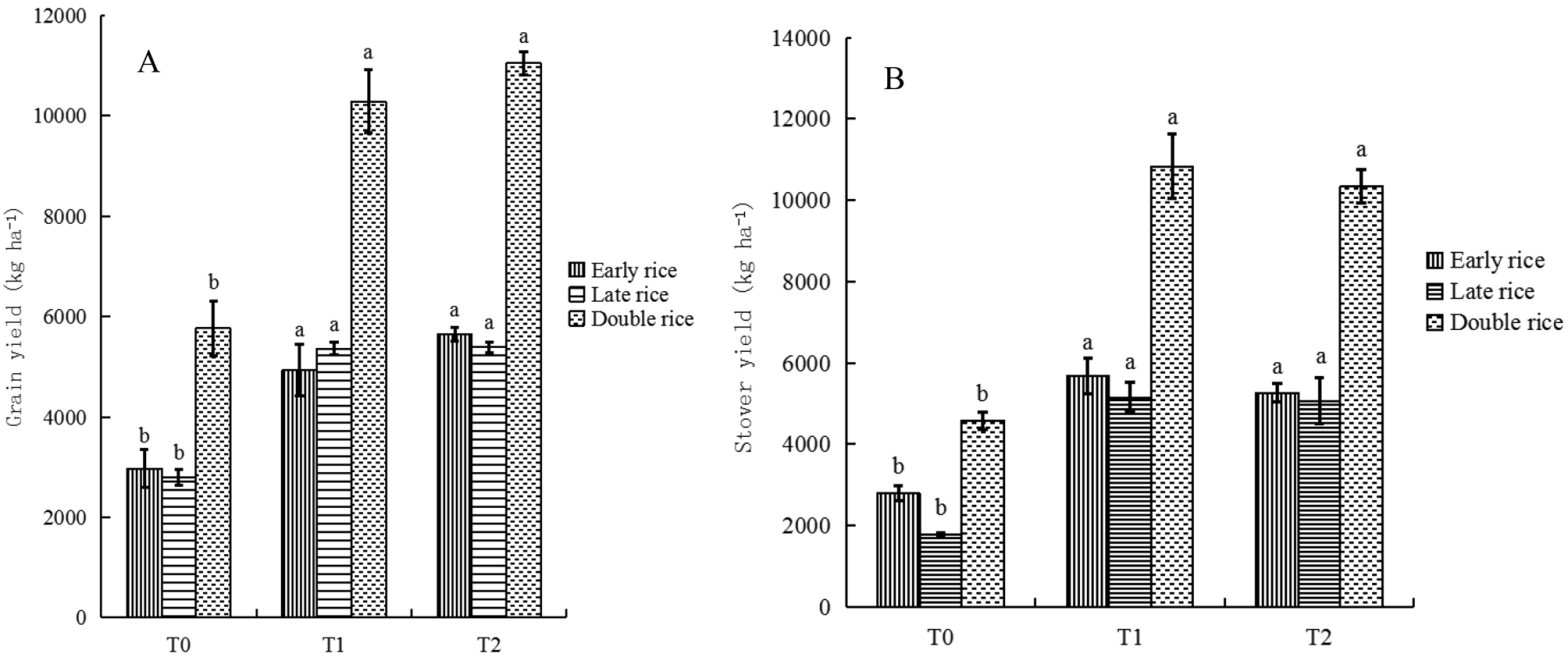
Grain (**A**) and stover (**B**) yield of rice as influenced by fertilization and irrigation in 2018. *Notes*: T_0_ = Traditional irrigation; T_1_ = Traditional irrigation and fertilization practice; T_2_ = Water-saving irrigation and optimizing fertilization. The different lower-case letters indicate significant differences between different treatments at *P *< 0.05 according to Duncan multiple range test. Error bars show SE (*n* = 3).

Rice grain yields in the T_2_ treatment increased by 0.92 times, stover yields increased by 1.26 times compared to those in the T_0_ treatment, respectively (*P* < 0.05) (Fig. 1A, B). However, there were no significant differences in the stover and grain yields between T_2_ and T_1_ treatments (Fig. 1A, B). Meanwhile, soil pH values varied between 5.97 and 6.24 under different fertilization and irrigation regimes, and thus the soils were acidic (Tables 1, 2). Meanwhile, soil pH and SOC content were significantly (*P* < 0.05) higher in the T_2_ treatment than those in the other two treatments. Soil NH_4_^+^-N content in the T_1_ and T_2_ treatments significantly (*P* < 0.05) increased compared with the T_0_ treatment. Additionally, there was a significant (*P* < 0.05) increase in soil NO_3_^-^-N content in the T_1_—treated soils compared to that in the T_0_—treated soils (Table 2). Nevertheless, no apparent differences occurred in the TN concentration in paddy soils among different fertilization and irrigation regimes. In addition, the PAO in the T_0_ treatment significantly (*P* < 0.05) decreased compared with that in the T_1_ and T_2_ treatments, but the PAO in the later two treatments was not significantly different (Table 2).

**Table 1:** Annual water and fertilizer input in Chinese double rice—cropping systems.

Treatments	Fertilization	Irrigation
T_0_	No chemical fertilization	Traditional flooding irrigation
T_1_	Conventional level of nitrogen (273 kg N ha^−1^), phosphorus (59 kg P ha^−1^), and potassium (112 kg K ha^−1^) fertilizer application	Traditional flooding irrigation
T_2_	Optimum level of nitrogen (240 kg N ha^−1^), phosphorus (52 kg P_2_O_5_ ha^−1^), and potassium (198 kg K_2_O ha^−1^) fertilizer application	Shallow intermittent irrigation

T_0_ = Traditional irrigation; T_1_ = Traditional irrigation and fertilization practice; T_2_ = Water-saving irrigation and optimizing fertilization.

**Table 2:** Soil chemical properties and potential ammonia oxidation as influenced by fertilization and irrigation in 2018.

Treatments	pH	SOC (g kg^-1^)	TN (g kg^-1^)	NO_3_^-^-N (mg kg^-1^)	NH_4_^+^-N (mg kg^-1^)	PAO (μg NO_2_^–^N g^-1^ d.w.day^-1^)
T_0_	5.97 ± 0.08b	15.16 ± 0.10b	2.18 ± 0.25a	13.20 ± 0.77b	35.14 ± 6.12b	0.004 ± 0.002b
T_1_	6.01 ± 0.08b	15.33 ± 0.13b	2.00 ± 0.12a	18.74 ± 2.94a	59.64 ± 5.53a	0.010 ± 0.002a
T_2_	6.24 ± 0.11a	15.98 ± 0.18a	1.84 ± 0.16a	6.37 ± 0.96c	51.55 ± 7.43a	0.011 ± 0.004a

T_0_ = Traditional irrigation; T_1_ = Traditional irrigation and fertilization practice; T_2_ = Water-saving irrigation and optimizing fertilization. SOC: soil organic carbon; N: nitrogen; TN: total N; NO_3_^-^—N: nitrate N; NH_4_^+^—N: ammonium N; PAO: potential ammonia oxidation. Values (means ± SD) with different lower-case letters in a column are significantly different at *P* < 0.05 according to the Duncan test.

### Soil ammonia-oxidizing archaea community

**Figure 2:**
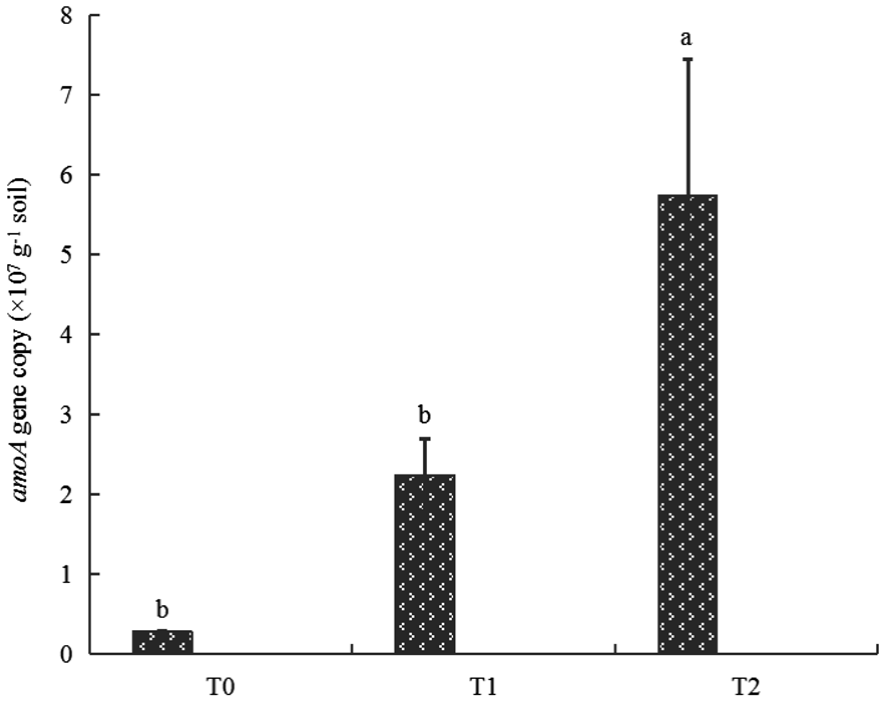
Abundances of soil ammonia-oxidizing archaeal *amoA* genes in response to different fertilization and irrigation. The different letters indicate significant differences (Duncan’s test, *P* < 0.05). *Notes*: T_0_ = Traditional irrigation; T_1_ = Traditional irrigation and fertilization practice; T_2_ = Water-saving irrigation and optimizing fertilization.

**Figure 3:**
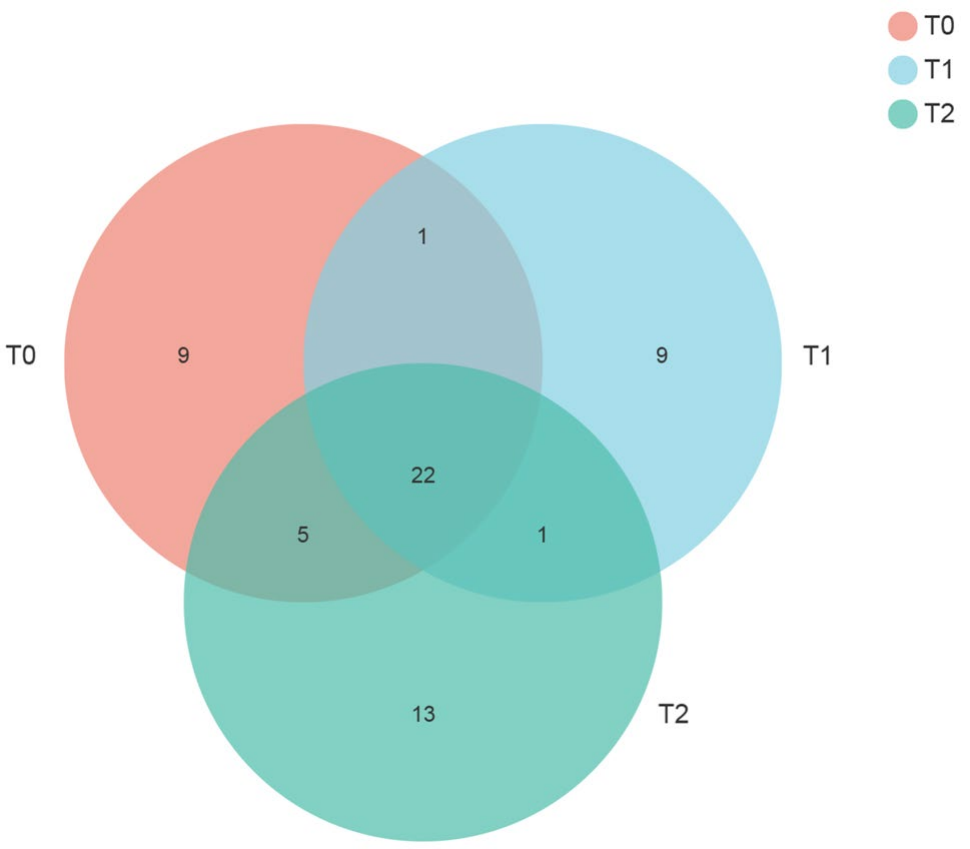
Venn diagram depicts operational taxonomic units (OTUs) of soil ammonia-oxidizing archaea (AOA) that were shared or unique for T_0_, T_1_, and T_2_. *Notes*: T_0_ = Traditional irrigation; T_1_ = Traditional irrigation and fertilization practice; T_2_ = Water-saving irrigation and optimizing fertilization.

**Figure 4:**
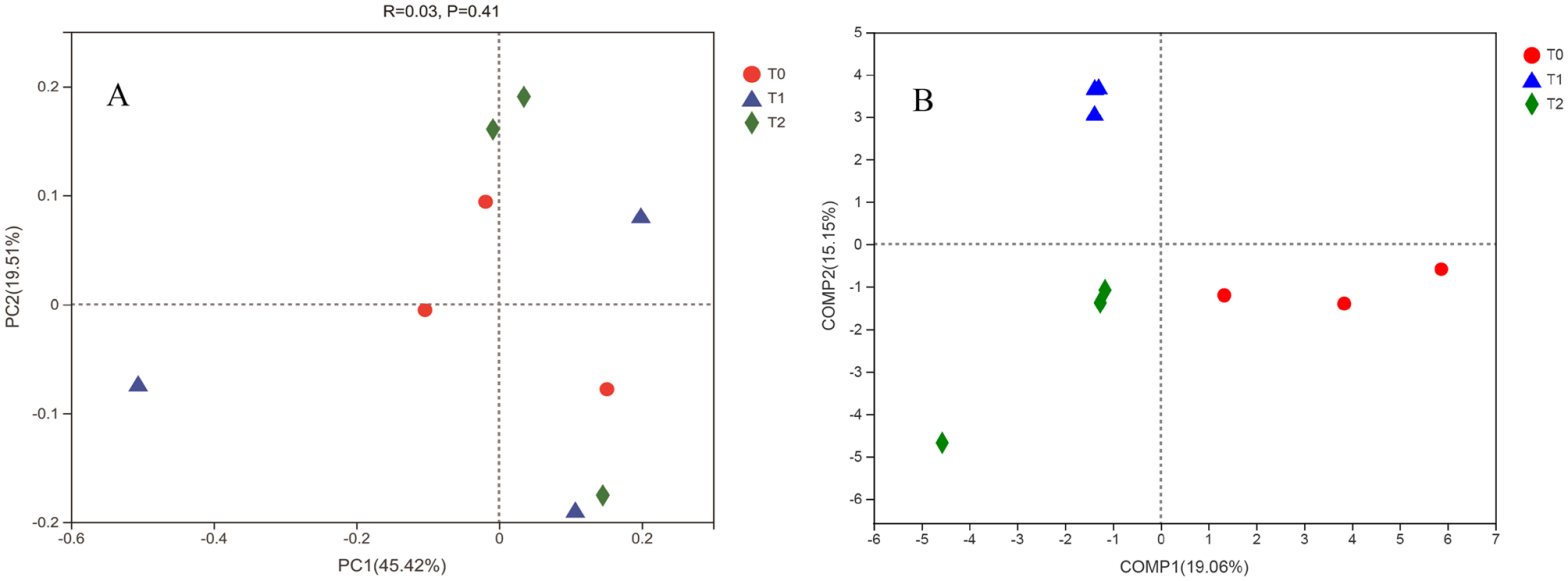
Comparison of the community structure of ammonia-oxidizing archaea (AOA) at the OTU level under different fertilization and irrigation regimes using principal coordinates analysis (PCoA) (A) and partial least squares discriminant analysis (PLS—DA ) (B) based on Bray–Curtis distance, respectively. *Notes*: *R* and *P* are from ANOSIM analysis comparing the beta diversity associated with T_0_, T_1_, and T_2_ treatments. T_0_ = Traditional irrigation; T_1_ = Traditional irrigation and fertilization practice; T_2_ = Water-saving irrigation and optimizing fertilization.

*AOA abundance and diversity*. In this study, AOA were the major groups of ammonia-oxidizers in the studied paddy soils, whereas the DNA band of AOB was too weak to detect by gel electrophoresis (Supplementary Figure S1). Meanwhile, the AOA abundance was estimated by quantifying their *amoA* gene copy numbers. In this study, the AOA *amoA* gene copy number (5.74 × 10^7^ *amoA* gene copies g^-1^ dry soil) was higher in the T_2_—treated soils than that in the other two treatments (*P* < 0.05) (Fig. 2). In addition, the rarefaction curves reached saturation, indicating that the generated sequences were enough to reflect the diversity of AOA *amoA* genes (Supplementary Figure S2). Furthermore, a query coverage was > 99.99%, suggesting that this study captured the dominant OTUs of AOA in each soil sample (Table 3). The Venn diagram indicated that the numbers of AOA OTUs at a 97% sequence identity were 37, 33, and 41 in the T_0_ -, T_1_—and T_2_—treated soils, respectively (Fig. 3). Only 36.67% of the total AOA OTUs were shared in three different soils treated by fertilization and irrigation (Fig. 3). Additionally, the proportion of shared OTUs in AOA between T_1_—and T_2_—treatments was 64.70% in their total sequences (Fig. 3). The alpha diversity of the AOA—related OTUs did not differ significantly (*P* > 0.05) among fertilization and irrigation regimes (Table 3), suggesting that it remained steady after water and fertilizer application in the paddy soil. No variations in the alpha diversity of AOA among the different treatments depended largely on the magnitude of soil pH change, which was an important factor affecting the AOA community in acidic soils^16^. In this study, only a marginal (0.27) difference in pH value between the T_0_, T_1_, and T_2_- treated soils was found, which might be not substantial enough to alter the alpha diversity of AOA for their adaptation to a wide range of pH^16^. Similarly, for beta diversity, there was no significant change in AOA community structure by PCoA with Bray–Curtis distance (Fig. 4A), irrespective of fertilization and irrigation regimes. Furthermore, ANOSIM tests confirmed no significant differences in the composition of the AOA community among the treatments (*R* = 0.03, *P* = 0.41) (Fig. 4A). However, the replicate samples T_01_, T_02_, and T_03_, the samples T_11_, T_12_, and T_13_, and the samples T_21_, T_22_, and T_23_ within the same treatment could not be grouped together (Fig. 4A), indicating that the experimental replication in the same treatment was not good in this field trial, possibly because of spatial variation in soil AOA community across field experimental plots. Under these circumstances, a supervised method, partial least squares discriminant analysis (PLS—DA), was chosen to maximize the variance among different soil samples, while minimizing the variance within each sample^17^. PLS—DA revealed that fertilization and irrigation treatments altered AOA community composition (Fig. 4B). A total 34.21% of the variations in the AOA community composition could be explained by the first two principal components (Fig. 4B).

**Table 3:** Diversity of soil ammonia-oxidizing archaea (AOA) as affected by fertilization and irrigation in 2018.

Treatments	OTUs	Coverage (%)	Richness		Diversity	
			ACE	Chao 1	H′	D
T_0_	16,573 ± 2812a	99.998 ± 0.433a	23 ± 3a	23 ± 3a	2.05 ± 0.18a	0.20 ± 0.05a
T_1_	17,049 ± 4413a	99.999 ± 0.266a	18 ± 5a	18 ± 4a	1.89 ± 0.40a	0.22 ± 0.10a
T_2_	17,956 ± 3252a	99.990 ± 0.456a	26 ± 3a	25 ± 2a	1.69 ± 0.17a	0.29 ± 0.07a

T_0_ = Traditional irrigation; T_1_ = Traditional irrigation and fertilization practice; T_2_ = Water-saving irrigation and optimizing fertilization. Operational taxonomic units (OTUs); Abundance-based coverage estimators (ACE); . Values (means ± SD) with different lower-case letters in a column are significantly different at *P* < 0.05 according to the Duncan test.

**Figure 5:**
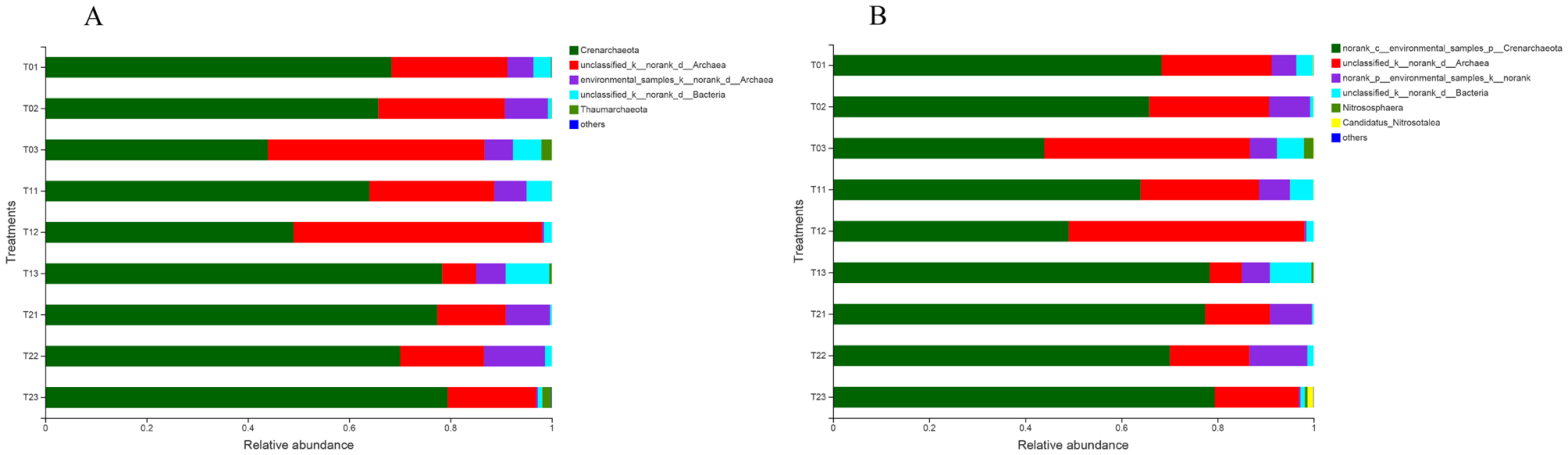
Relative abundance of ammonia-oxidizing archaea (AOA) phyla (**A**) and genera (**B**) under different fertilization and irrigation regimes. The relative abundance is expressed as the mean percentage of the targeted sequences to the total high-quality bacterial and fungal sequences of samples from triplicate plots of each irrigation and fertilization treatment. *Notes*: T_0_ = Traditional irrigation; T_1_ = Traditional irrigation and fertilization practice; T_2_ = Water-saving irrigation and optimizing fertilization.

*AOA community composition*. A total of 154,733 AOA sequence reads, ranging from 12,837 to 21,639 per soil sample after QIIME quality filtering, were taxonomically classified into five phyla and six genera (Fig. 5A, B). The dominant AOA phylum in paddy soils was *Crenarchaeota* (the sequence number at the phylum level varied from 59.36% to 75.62% in soils), whereas the rare AOA phylum was characterized by low *Thaumarchaeota* (Fig. 5A). Moreover, T_2_ resulted in the prevalence of the AOA phylum *Crenarchaeota*, which accounted for 75.62% of the total AOA (Fig. 5A). However, T_1_ decreased the relative abundance of the phylum *Thaumarchaeota* by 78.38% and 73.33%, respectively, compared to that in the T_0_ and T_2_ treatments (Fig. 5A). Meanwhile, the T_1_ and T_2_ treatments decreased the abundance of the genus *Nitrososphaera* by 77.62% and 70.15% compared with that in the T_0_ treatment, respectively (Fig. 5B).

**Figure 6:**
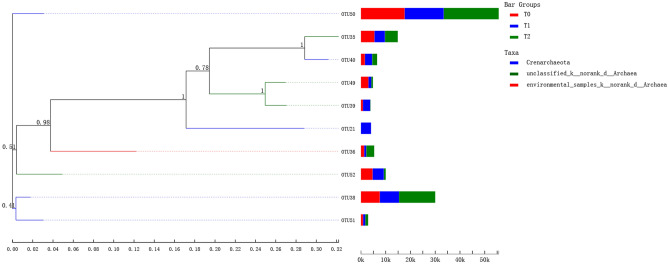
Neighbor-joining tree for representative ammonia-oxidizing archaea (AOA) OTUs (representatives with relative abundance of the top 10 most abundant species). Bootstrap values (> 50%) were indicated at branch points. OTUs from this study were shown in bold. *Notes*: T_0_ = Traditional irrigation; T_1_ = Traditional irrigation and fertilization practice; T_2_ = Water-saving irrigation and optimizing fertilization.

Phylogenetic tree for the *amoA* gene of AOA was constructed based on the values of OTUs of the top 10 most abundant species, which indicated that most of AOA OTUs were affiliated with *Crenarchaeota* cluster and unclassified_k__norank_d__*Archaea* cluster, accounting for 64.12% and 21.76% of total reads, respectively (Fig. 6). In addition, the OTUs 21, 40, and 50 belonged to cluster *Crenarchaeota*, and the OTUs 35, 39, 49, and 52 for cluster unclassified_k__norank_d__*Archaea*, respectively (Fig. 6).

### Relationships between ammonia-oxidizing archaeal communities and edaphic characteristics

**Figure 7:**
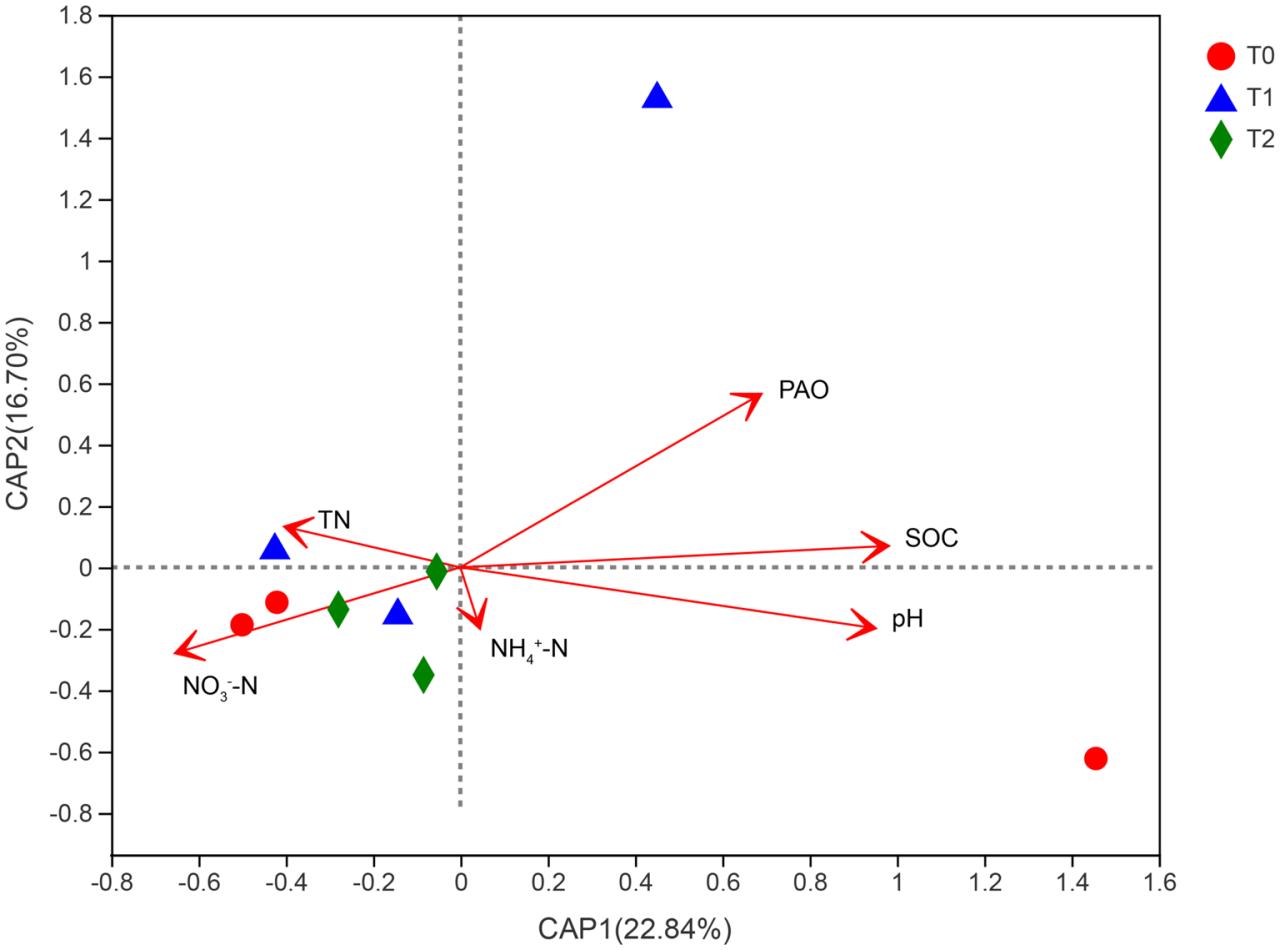
Distance-based redundancy analysis (db-RDA) of ammonia-oxidizing archaea (AOA) communities according to edaphic factors. *Notes*: T_0_ = Traditional irrigation; T_1_ = Traditional irrigation and fertilization practice; T_2_ = Water—saving irrigation and optimizing fertilization. SOC: soil organic carbon; N: nitrogen; TN: total N; NO_3_^-^—N: nitrate N; NH_4_^+^—N: ammonium N; PAO: potential ammonia oxidation.

**Figure 8:**
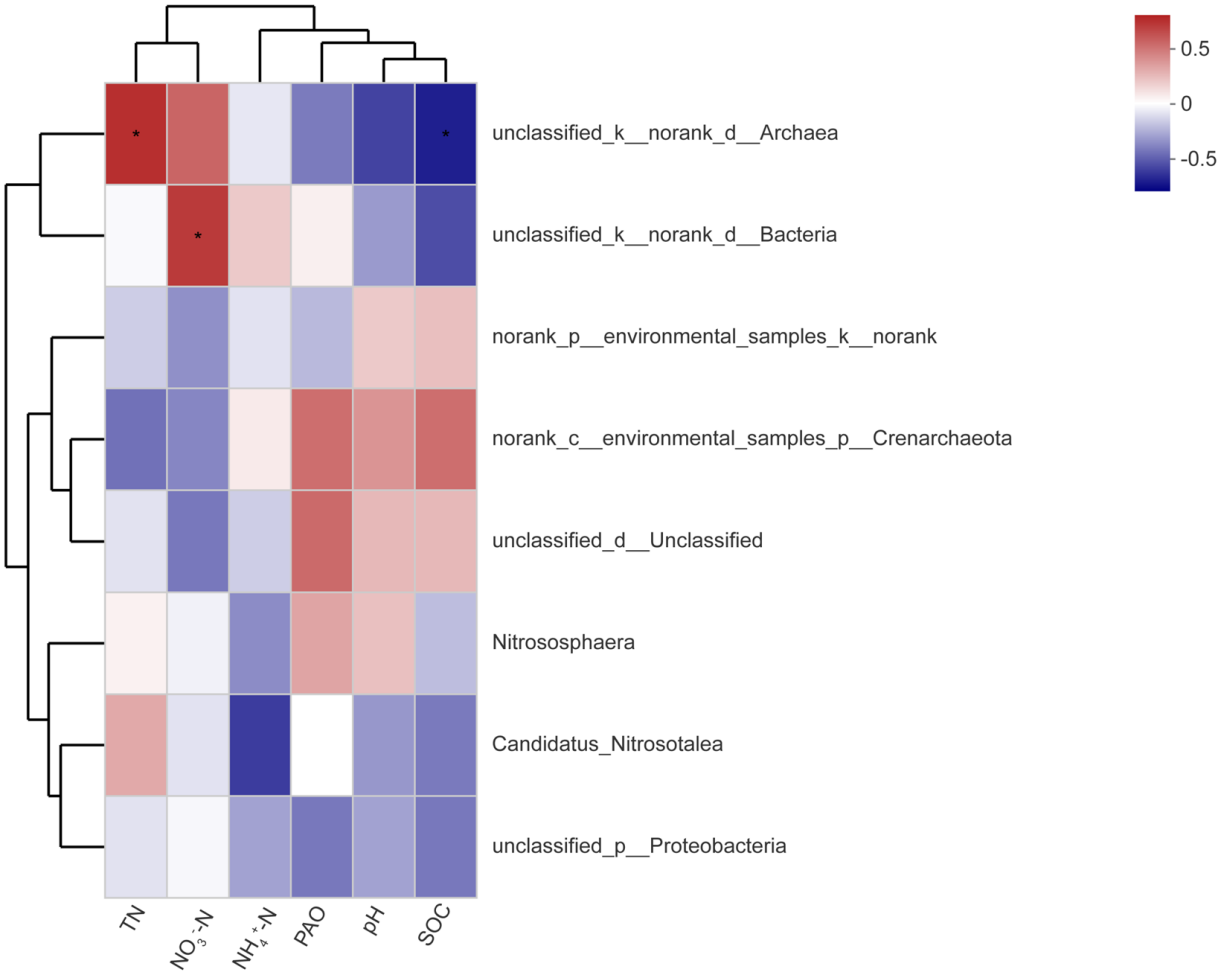
Correlation heatmap of edaphic characteristics and relative genus abundances of ammonia-oxidizing archaea (AOA). The color intensity expresses the correlative values of edaphic characteristics with relative genus abundances. ^*^P < 0.05. *Notes*: T_0_ = Traditional irrigation; T_1_ = Traditional irrigation and fertilization practice; T_2_ = Water-saving irrigation and optimizing fertilization. SOC: soil organic carbon; N: nitrogen; TN: total N; NO_3_^-^—N: nitrate N; NH_4_^+^—N: ammonium N; PAO: potential ammonia oxidation.

Correlation analysis showed that the AOA abundance had positive correlations with soil pH (*r* = 0.974, *P* < 0.01), SOC (*r* = 0.986, *P* < 0.01), and PAO (*r* = 0.852, *P* < 0.01), but had negative relationships with TN (*r* = -0.982, *P* < 0.01) and NO_3_-N (*r* = -0.677, *P* < 0.05) contents (Table 4). Furthermore, soil pH (*r*^2^ = 0.670, *P* = 0.03), SOC content (*r*^2^ = 0.688, *P* = 0.012), and PAO (*r*^2^ = 0.569, *P* = 0.048) were significantly (*P* < 0.05) positively correlated with the AOA community structure by a db—RDA (Fig. 7). The first ordination db—RDA axis (axis 1, horizontal), which was strongly related to pH and SOC, explained 22.84% of the total variability in the AOA community structure. The second ordination db—RDA axis (axis 2, vertical) was mainly related to PAO, explained 16.70% of the variability in the AOA community structure (Fig. 7). Meanwhile, the relative abundance of the genus unclassified_k__norank_d__*Archaea* was significantly (*P* < 0.05) positively related to TN content, but was significantly (*P* < 0.05) negatively correlated with SOC content (Fig. 8).

**Table 4:** Correlation coefficients between soil properties and the abundance of soil ammonia-oxidizing archaea (AOA).

Item	pH	SOC	TN	NH_4_^+^-N	NO_3_^–^N	PAO
AOA abundance	0.974**	0.986**	-0.982**	0.529	-0.677*	0.852**

SOC: soil organic carbon; N: nitrogen;TN: total N; NO_3_^-^—N: nitrate N; NH_4_^+^—N: ammonium N; PAO: potential ammonia oxidation. *P < 0.05; **P < 0.01.

## Discussion

The abundance of AOA had richer than that of AOB in acidic paddy soils under different irrigation and fertilization regimes (Supplementary Figure S1). A lower abundance of AOB in the same type of rice paddy soil has also been reported in other studies^3,18^, because AOA could have a potentially competitive advantage over AOB in acidic and oligotrophic soil environments. Furthermore, AOA were more stable and resilient than AOB under different environmental conditions^19^.

The AOA abundance was reflected by the AOA *amoA* gene copy number. In this study, the AOA *amoA* gene copy number significantly (*P* < 0.05) increased in the T_2_—treated soil, but no apparent difference occurred in the T_1_—treated soil, in comparison with that in the T_0_—treated soil (Fig. 2). This finding demonstrated that the growth of AOA communities was significantly stimulated by the optimized fertilizer and water management practice. However, this result did not support the hypotheses that the oligotrophic nature of AOA makes them important for nutrient-limited soil ecosystems^14,20^. Additionally, these findings were inconsistent with those reported by Fang et al. (2019), who found that there was no obvious difference in the AOA *amoA* gene copy number between the NPK and CK treatments^10^. Furthermore, the AOA *amoA* gene copy number ranged from 0.28 × 10^7^ to 5.74 × 10^7^ per gram dry soil (Fig. 2), which was about tenfold higher than the value obtained by Fang et al. (2019)^10^. These inconsistent results mainly depended on soil types, rice cultivars, and agricultural practices^11,15,21^. In summary, a number of factors could be responsible for shaping the abundance of AOA *amoA* genes under different environmental conditions.

This study also indicated that fertilization and irrigation treatments formed different AOA community compositions based on PLS—DA (Fig. 4B). This agreed with a previous report showing the proper combination of fertilization rate and irrigation frequency could regulate soil AOA community composition^11^. In addition, the most predominant OTUs of AOA had affiliation to *Crenarchaeota* (Fig. 6), which differed from the previous findings indicating that the dominant OTUs of AOA fell into *Nitrososphaera* in many soil systems^10,15^. The difference in AOA community structure in different types of soils reveals a separation and selectivity of AOA induced by their own growth characteristics and habitat conditions. Moreover, these findings indicated that AOA members were dominated by distinct AOA species under different environmental conditions^15,22^. In comparison with the other two treatments, the T_2_ treatment increased the abundances of the phylum *Crenarchaeota* in soils. The members of the phylum *Crenarchaeota* are widely distributed in terrestrial and aquatic environments. Furthermore, *Crenarchaeota* are important players in the nitrogen cycle^23^. In general, these findings indicated a possible niche differentiation for AOA populations in which different soil microsites supported different AOA species.

The changes in AOA abundance and community composition, in turn could result in altered rates and/or controls of corresponding functions. This study suggested that the AOA-specific PAO in the T_1_- and T_2_-treated soils was significantly (*P* < 0.05) greater than that in the T_0_-treated soils (Table 2). The result is similar to that obtained by previous studies indicating that an enhanced PAO in soils was observed in the NPK treatments compared to that without fertilizer application^10^. This supported the hypothesis that an increase in the AOA abundance could enhance PAO in soils^24^, as confirmed in this study (Table 4). The stimulation of PAO might increase N availability for rice plants and thus resulted in higher rice yields^10,15^, as evidenced in Fig. 1A. However, different fertilization and irrigation treatments had a small impact on the alpha diversity of AOA in paddy soils (Table 3). The reasons for nonsignificant effects of fertilization and irrigation treatments on the alpha diversity might be closely related to their pH adaptation. AOA could be clustered into acidophilic, acidoneutral, and alkalinophilic groups based on a pH gradient, with the former dominant in soils with pH < 5.5, acido-neutral AOA dominant in soils with pH 5.5–7.5, and the latter dominant in soils with pH > 7.5^16,22,25^. In this study, the soil samples among all treatments had the relatively narrow pH range of 5.97–6.27; thus, soil pH had no significant impact on the AOA alpha diversity. Taken together, this study indicated that fertilization and irrigation could influence the abundance, activity and community structure of AOA rather than their alpha diversity in the paddy soil.

The variation in the AOA community structure and abundance might be due to their responses to altered edaphic properties associated with different fertilization and irrigation managements. A correlation analysis revealed that the AOA abundance was negatively related to TN and NO_3_^–^N contents in the acidic paddy soil (*P* < 0.05) (Table 4). Conversely, positive correlations existed between the AOA abundance and TN, NH_4_^+^-N, and NO_3_^–^N contents^10^. The inconsistent findings were perhaps because of different sampling times^15^. Meanwhile, the AOA abundance was positively correlated with SOC content in the paddy soil (*P* < 0.01) (Table 4). Likewise, Zhang et al. (2013) reported that the AOA abundance had a significant positive relationship with SOC content, which supported the idea of heterotrophic growth of the AOA community^26^. Additionally, the AOA abundance was positively related to pH (*P* < 0.01) (Table 4). However, the opposite results were previously obtained by Gao et al. (2018) and Fang et al. (2019), who found that there was a negative relationship between soil pH with the AOA abundance^3,10^. Additionally, pH also showed no significant correlations with the AOA abundance in alkaline soils^27^. These inconsistent results indicated that there were correlative variations in the AOA abundance and pH value in response to soil acidity-alkalinity gradients. In addition, soil pH (*r*^2^ = 0.67, *P* = 0.03) and SOC (*r*^2^ = 0.69, *P* = 0.01) were the most predominant edaphic factors for the AOA community structure under different fertilization and irrigation regimes by db—RDA (Fig. 7). Nonetheless, Fang et al. (2019) found that soil pH was nonsignificantly correlated with the AOA community composition^10^. The distinct responses of the AOA populations to soil pH were perhaps because these AOA communities grew well in their own narrow pH ranges^28^. Besides directly pH-selecting for acidophilic or neutrophilic AOA, pH could also influence soil nutrient availability, which indirectly mediated the AOA community composition. It has been reported that SOC, especially dissolved organic C (DOC) was another important edaphic factor driving the AOA community structure in acid soils^28^. In general, these studies further demonstrated that the abundance and community composition of AOA were shifted by altered soil properties associated with different fertilization and irrigation treatments, which might be the important factors responsible for variations in PAO.

## Conclusions

Our results showed that AOA (ammonia-oxidizing archaea) played the vital role in ammonia oxidation in acid paddy soils. The T_2_ (optimal irrigation and fertilization) treatment increased the AOA *amoA* gene copy number. Additionally, *Crenarchaeota* was the dominant phylum of AOA communities. Furthermore, an increase in the abundance of the phylum *Crenarchaeota* was observed in the T_2_ treatment compared with that in the other two treatments. Meanwhile, SOC (soil organic C), pH, and PAO (potential ammonia oxidation) were the highest in the T_2_—treated soils. The T_2_—treated soils had clear differences in the AOA community composition compared with that in the T_0_ (traditional irrigation)—and T_1_ (traditional irrigation and fertilization)—treated soils. The AOA community composition were mainly driven by soil pH and SOC. In addition, the AOA abundance showed a significant positive relationship with PAO. Furthermore, the T_2_ treatment increased rice yield compared to the T_0_ and T_1_ treatments. Overall, these results demonstrate that the T_2_ treatment may mediate the AOA community structure, promote ammonia oxidation rate, improve soil nutrient availability and thus maintain rice yield in the present study.

## Materials and methods

### Experiment design and sample collection

Experimental and field studies on rice plants complied with relevant Chinese guidelines^29,30^. A fertilization and irrigation experiment was established in 2008 and double cropping rice (*Oryza sativa* L.) was planted annually at Baisha Experimental Station (119°04′10″E, 26°13′31″N), Minhou County, Fuzhou City, Fujian Province, China. The early and late cultivars of rice are conventional rice varieties 78–30 and 428, respectively, from south China. This region has a subtropical monsoonal climate with an average annual temperature of 19.5 ℃ and mean annual precipitation of 1 350 mm. The soil is a typic Hapli-Stagnic Anthrosol (USDA soil system). At the beginning of the experiment, the soil had a pH 6.19, 14.16 g kg^-1^ soil organic matter (SOM), 0.66 g kg^-1^ total N (TN), 0.30 g kg^-1^ total P (TP), 3.8 mg kg^-1^ NO_3_-N, 12 mg kg^-1^ NH_4_^+^-N, 3.358 and 0.83 mg kg^-1^ of available P (AP) and K (AK), respectively, in the 0 − 20 cm soil layer. A randomized complete block design with three treatments was conducted in triplicate in 9 plots (4.0 m long × 5.0 m wide). Three treatments included control (no fertilization with traditional irrigation, T_0_), traditional fertilization with traditional irrigation (T_1_, based on local practices), and optimum fertilization with water-saving irrigation (T_2_, based on both fertilizer recommendation from local agriculture committee and water saving by shallow intermittent irrigation). The water and fertilizer practices used are described in Table 1. Conventional fertilization in this experiment was conducted with 273 kg N, 59 kg P, and 112 kg K ha^−1^, whereas optimum fertilization with 240 kg N, 52 kg P, and 198 kg K ha^−1^ for both the early and late rice crops. The fertilizers were urea (46% N), superphosphate (5% P), potassium chloride (50% K), and chemical compound fertilizer (15% N, 7% P, 12% K), respectively. The 100% of the total amount of P, 60% of N, and 40% of K fertilizers were used as basal fertilizers before transplanting of rice seedlings, and the 40% N and 60% K fertilizers as topdressing fertilizers after tillering, respectively (Table 1). Traditional irrigation was needed to be maintained at a depth of 1.0 − 6.0 cm during the rice-growing season, and water-saving irrigation at a depth of -3.0 to 3.0 cm in the field. To avoid water and fertilizer exchange between adjacent experimental units, a cement concrete border, with dimensions (length × width × height) 40 cm × 30 cm × 20 cm, was constructed. The early rice was transplanted with a 20.0 cm × 23.0 cm row spacing on 21 April and harvested on 25 July 2018. The late rice was transplanted with the same row spacing on 30 July and harvested on 1 December 2018.

After the rice harvest, the rice straw and grain were sampled separately to measure their yields in each plot in both the early and late seasons. The fractions from the above-ground parts of rice plants were oven-dried at 105 ℃ for 30 min, and then dried at 60 ℃ to a constant weight. The rice stover and grain yields were recorded, respectively^30^. In addition, five samples of 0 − 20 cm soil layer were collected and mixed from each plot after late rice harvest. The fresh soil samples were transported immediately on ice to the laboratory. Plant residues and stones were manually removed from soil samples. The soil samples were then mixed and sieved to < 2.0 mm. One subsample was stored at—80 ℃ for soil microbial analysis, while the other subsample was air-dried for chemical analysis.

### Plant, soil physiochemical properties, and potential ammonia oxidation (PAO)

Soil moisture was calculated as the difference between oven—dry (24 h at 105 ℃) and fresh weight. Soil pH was analyzed with a pH meter (Mettler Toledo, Greifensee, Switzerland) in a 1:2.5 (m:v) soil—water suspension. The SOC content was measured by means of the oxidation–reduction titration. The TN content was analyzed by using a Kjeldahl digestion. Both NH_4_^+^-N and NO_3_^−^-N in fresh soils were extracted with 2 M KCl (1:10 (m:v) soil/extract), and the extract was analyzed by UV spectrophotometry^31^. The PAO activity was measured according to Kurola et al. (2005)^32^ with minor modifications. Briefly, 5 g of fresh soil was incubated in 20 mL phosphate-buffered saline (PBS) and 1 mM of (NH_4_)_2_SO_4_ at room temperature in the dark for 24 h. Then 10 mg L^−1^ of KClO_3_ addition inhibited nitrite oxidation. At the end of incubation, soil NO_2_^−^-N was extracted with 5 mL of 2 M KCl. The optical density (at 540 nm) of the supernatant was determined after each centrifugation in order to calculate the NO_2_^–^N content by using sulfonamide and naphthylethylene diamide as reagents. The PAO activity was estimated by the slope of the NO_2_^–^N accumulation.

### Abundance and diversity of ammonia-oxidizing archaea

DNA was extracted from 0.25 g of fresh soils per sample by an E.Z.N.A Soil DNA Kit (Omega Bio-tek, Norcross, GA, USA) on the basis of the manufacturer’s instructions^33^. The DNA purity and concentration were detected by using a NanoDrop 2000 spectrophotometer (Thermo Fisher Scientific, Waltham, MA, USA).

The AOA *amoA* gene was amplified by the primers Arch-*amoA*F (5′ STAATGGTCTGGCTTAGACG 3′) and Arch-*amoA*R (5′GCGGCCATCCATCTGTATGT 3′)^34^. Base on the preliminary experiment, the reaction systems and cycling conditions such as DNA amounts, annealling temperatures, and circular times were further optimized. The preliminary PCR amplification was performed for 27 cycles. All PCR products after 2.0% (w/v) agarose gel electrophoresis on a 2.0% (w/v) agarose gel is used to verify the size of the target DNA for AOB and AOA. The band numbers and relative intensities of PCR products were analyzed by using Quantity One analysis software (Bio-Rad). However, only the AOA community was found and further analyzed under the following conditions (Supplementary Figure S1). Each 20-μL qPCR reaction mixture contained 10 μL 2X Taq Plus Master Mix (VazymeBiotech, Nanjing, China), 0.8 μL forward and reverse primers (5 μM), 1 μL DNA template and 7.4 μL ddH_2_O. The qPCR of AOA *amoA* gene was conducted on an ABI 7300 thermocycler (Applied Biosystems, California, USA) at 95 °C for 5 min, 95 °C for 30 s, 60 °C for 30 s, and 72 °C for 1 min. The purified PCR products were ligated into the plasmid pMD19-T vector (Takara, Dalian, China) carrying AOA *amoA* insert. Individual clones were grown in Luria–Bertani (LB) medium at 37 °C for 18 h. Plasmid DNA from a 5-mL culture was extracted with a TIANprep Mini Plasmid Kit (Tiangen, Beijing, China) and quantified by a NanoDrop 2000 UV–Vis spectrophotometer. Each sample and each standard was quantified in triplicate. The qPCR was performed with three times, and the amplification efficiency of the qPCR was 89.09% (*R*^2^ = 0.9998) for AOA. The cell number of AOA was calculated from the quantified number of the *amoA* gene on the basis of that each cell in AOA contains one copy of the *amoA* genes^35^.

The primers Arch-*amoA*F and Arch-*amoA*R^34^ were used to amplify *amoA* gene fragments by a GeneAmp PCR system 9700 thermocycler (Applied Biosystems, Foster City, CA, USA). The PCR reactions for AOA were performed with the following conditions: denaturation at 95 °C for 3 min , 27 cycles of 95 °C for 30 s, annealing at 55 °C for 30 s, elongation at 72 °C for 45 s, and a final extension at 72 °C for 10 min. The PCR reaction mixture (20 μL) contained 4 μL of 5 × FastPfu Buffer, 2 μL of 2.5 mM dNTPs, 0.8 μL of 5 μM each primer, 0.4 μL of FastPfu Polymerase, and 10 ng of template DNA. The PCR products were run on 2% agarose gels and purified with an AxyPrep DNA Gel Extraction Kit (Axygen, USA), and quantified by using QuantiFluor-ST (Promega Corporation, Madison, WI, USA) according to the manufacturer’s instructions. The purified amplicons were pooled in equimolar concentrations and paired-end sequenced (2 × 300) by an Illumina MiSeq platform (Illumina, San Diego, CA, USA) on the basis of the standard protocols by the Majorbio Bio-Pharm Technology Co. Ltd. (Shanghai, China).

Raw fastq files were demultiplexed, filtered by Trimmomatic software (version 3.29) and merged by FLASH (version 1.2.7) with the criteria: (i) The sequences were trimmed at any site receiving an average quality score < 20 over a 50 bp sliding window. (ii) Sequences with mismatches to either the primer (> 2) or with ambiguous bases (> 1) were discarded. (iii) Sequences with their overlap > 10 bp or length < 200 bp were deleted. The remaining sequences were selected for chimeras by UCHIME^36^. The high-quality sequences were assigned to operational taxonomic units (OTUs) according to 97% sequence identity by the UPARSE pipeline. The taxonomy of each *amoA* gene sequence was identified by the Ribosomal Database Project (RDP) Classifier tool (http://rdp.cme.msu.edu/) against the fgr/*amoA* database (GeneBank Release 7.3, http://fungene.cme.msu.edu) with a confidence threshold of 70%. Furthermore, the rarefaction curve and other OTUs-based parameters, including coverage estimators (ACE), Chao1, Shannon–Wiener index (H′), and Simpson's index (D) were analyzed by the mothur software package^37^. Chao1 and ACE were used to evaluate the AOA community richness on the basis of the degree of sequence dissimilarity. H′ and D were used to evaluate to the alpha diversity within each individual sample^38^. In addition, the number of shared and unique AOA OTUs among three treatments was described by a Venn diagram. Furthermore, principal coordinates analysis (PCoA) and analysis of similarity (ANOSIM) with Bray–Curtis distance, and PLS—DA were used to evaluate beta diversity, in order to explore similarities and differences in AOA community structure. A heatmap analysis was performed to compare the relative abundance of the top 10 AOA genera. Moreover, a heatmap of relationship between the relative genus abundances of AOA and soil properties (e.g., pH, SOC, and TN) was conducted by Canoco for Windows 4.5 package. In addition, environmental factors were selected by the functions of envfit (permu = 999) and variance inflation factor (vif).cca, and the environmental factors such as SOC, TN, NH_4_^+^–N, NO_3_^-^–N, and PAO with vif < 10 were retained. The distance—based redundancy analysis (db‐RDA) was processed by *R* software (version 3.3.1). The phylogenetic analysis on the basis of the sequences acquired from this study and reference sequences from the NCBI GenBank was made by the molecular evolutionary genetics analysis (MEGA) software version 10.0 to construct a phylogenetic tree by the neighbor-joining method^39^. All bioinformatics analyses for soil AOA communities were performed on online “I-Sanger” (http://www.i-sanger.com/) developed by Shanghai Majorbio Bio-Pharm Technology Co., Ltd. All original nucleotide sequence reads were deposited at the NCBI Sequence Read Archive (SRA) with the accession number of SRP293735.

One-way analysis of variance (ANOVA) and Duncan's multiple range tests were used to estimate the statistical significance of the differences of edaphic characteristics, rice yields, AOA *amoA* gene abundance and alpha-diversity among different water and fertilizer regimes by SAS version 8.02 (SAS Institute Inc., Carey, North Carolina, USA). All data were expressed as mean ± SD (*n* = 3).

## Supplementary Information

Supplementary Information.
