# Supplementary material for: Soil ammonia-oxidizing archaea in a paddy field with different irrigation and fertilization managements
Source: Sci Rep. 2021 Jul 15;11:14563. doi: 10.1038/s41598-021-93898-y (PMC8282617; doi:10.1038/s41598-021-93898-y)
Supplement: Supplementary file 1 — Supplementary Information. [file 41598_2021_93898_MOESM1_ESM.doc]

**
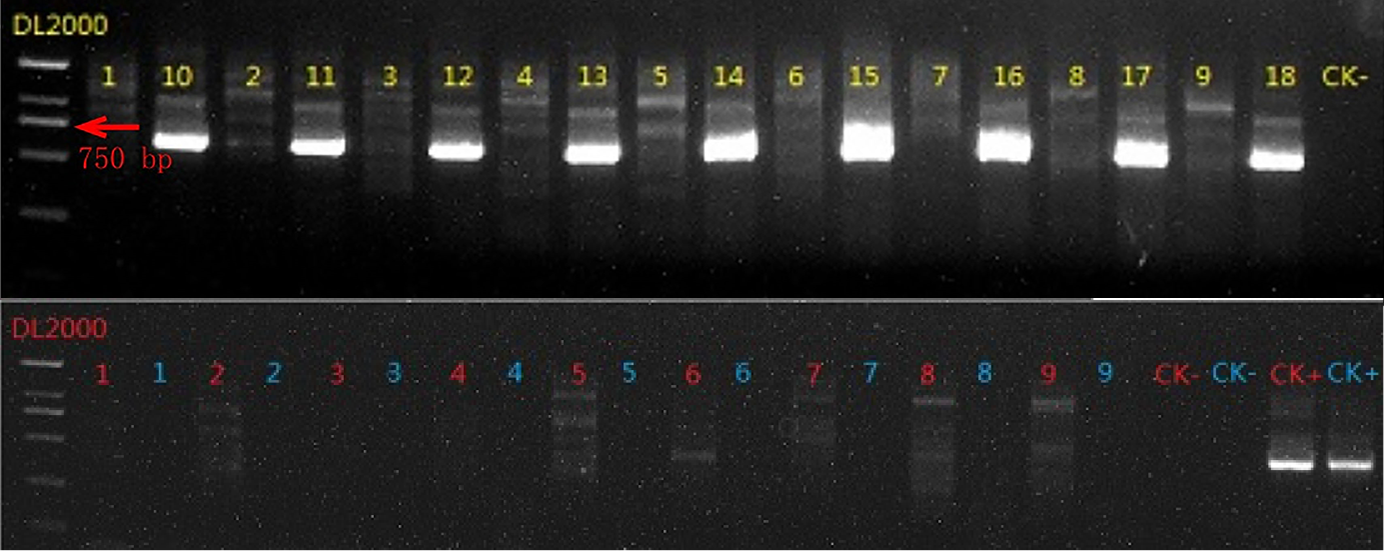
**

**Supplementary Figure 1.** **Electrophoretic analysis of soil ammonia-oxidizing bacteria (AOB) and ammonia-oxidizing archaea (AOA) as influenced by irrigation and fertilization in 2018.** Notes: T_0_ (1, 4, 7, 10, 13, 16) = Traditional irrigation; T_1_ (2, 5, 8, 11, 14, 17) = Traditional irrigation and fertilization practice; T_2_ (3, 6, 9, 12, 15, 18) = Water-saving irrigation and optimizing fertilization. The bands (1 - 9) in the electrophoretogram of PCR product was used as an analysis of AOB, and the bands (10 - 18) used as AOA under different reaction systems and cycling conditions. Different PCR reaction conditions were denoted by yellow, red, and blue fonts, respectively. CK-: sterile water, negative control; CK+: AOB *amoA* gene, positive control.


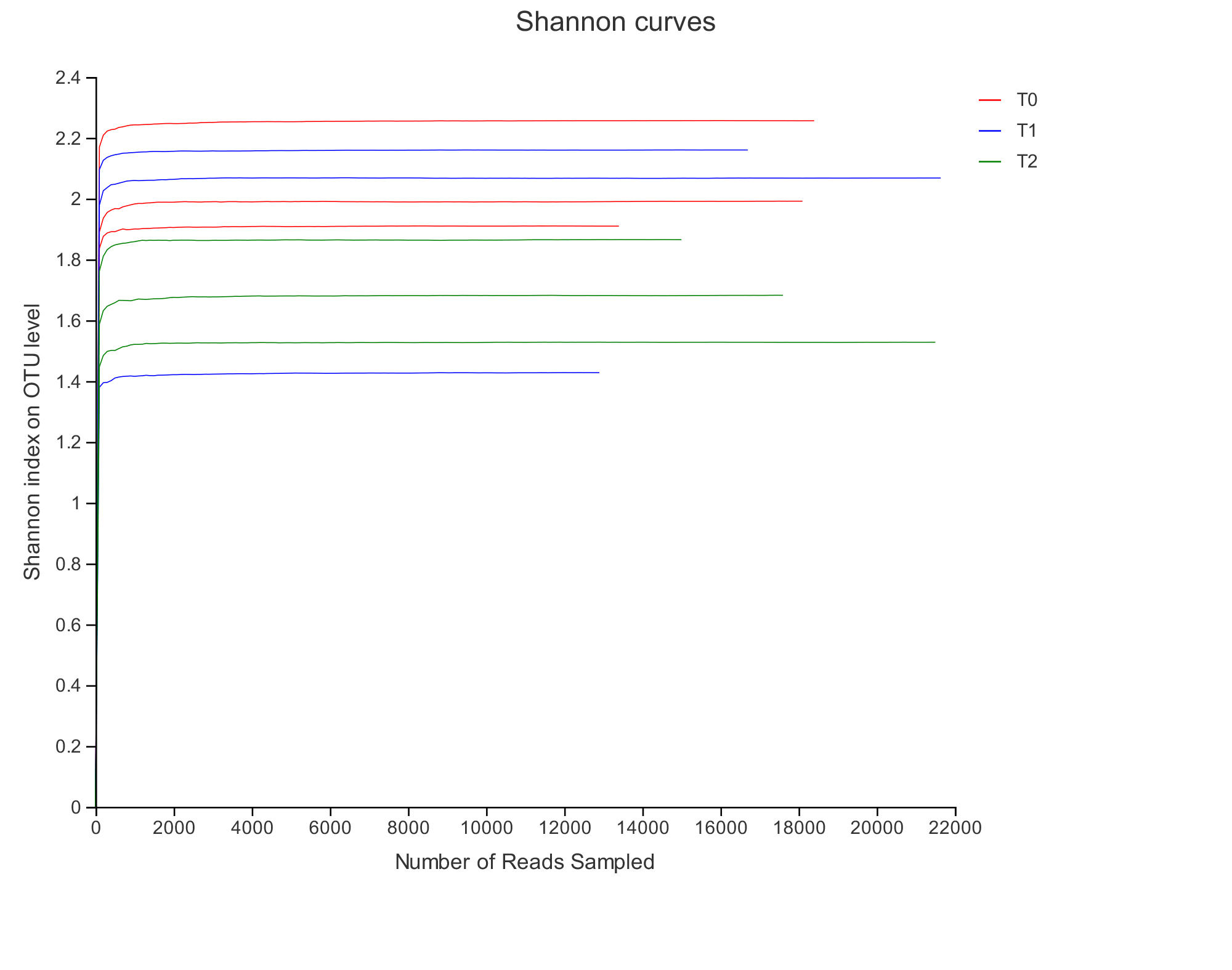


**Supplementary Figure 2. Shannon-Wiener curves of soil ammonia-oxidizing archaea (AOA) for the normalized number of reads at the 97% level of similarity as influenced by fertilization and irrigation in 2018.** Notes: T_0_ = Traditional irrigation; T_1_ = Traditional irrigation and fertilization practice; T_2_ = Water-saving irrigation and optimizing fertilization; Operational taxonomic unit (OTU).
